# Supplementary material for: Structural insights into binding-site access and ligand recognition by human ABCB1
Source: EMBO J. 2025 Jan 13;44(4):991–1006. doi: 10.1038/s44318-025-00361-z (PMC11833089; doi:10.1038/s44318-025-00361-z)
Supplement: Supplementary file 1 — Table EV1 [file 44318_2025_361_MOESM1_ESM.docx]

| **Table EV 1: Ion filtering and charge assignment parameters** | |
| --- | --- |
| **Ion Filtering** | |
| R^2^ Threshold | 0.996 |
| Duration threshold | 0.42 |
| Minimum Time of Death | 0.2 |
| Maximum Time of Birth | 0.1 |
| Signal-To-Noise Threshold | 3 |
| **Charge Assignment (Voting v3)** | |
| Bin Size (ppm) | 5 |
| Minimum Ions in Bin | 1 |
| Number of Charge Neighbors | 2 |
| Number of Isotope Neighbors | 5 |
