# Supplementary material for: Structural insights into binding-site access and ligand recognition by human ABCB1
Source: EMBO J. 2025 Jan 13;44(4):991–1006. doi: 10.1038/s44318-025-00361-z (PMC11833089; doi:10.1038/s44318-025-00361-z)
Supplement: Supplementary file 2 — Table EV2 [file 44318_2025_361_MOESM2_ESM.docx]

**Table EV2: Cryo-EM data collection, refinement and validation statistics of human ABCB1.** For the ATP-free ABCB1 complexes with zosuquidar and Taxol, only associated EM maps have been deposited and, consequently no model building and refinement statistics have been included.

| **Data collection and processing** | ABCB1_apo_ | ABCB1_Taxol/ATP_ | ABCB1  _Zosuquidar/ATP_ | ABCB1_ATP𝞬S_ | ABCB1_Zosuquidar_ | ABCB1_Taxol_ |
| --- | --- | --- | --- | --- | --- | --- |
| Microscope | FEI Titan Krios | | | | | |
| Camera | Gatan Biocontinuum K3 | | | | | |
| Magnification | 130kx | | | | | |
| Voltage (kV) | 300 | | | | | |
| Electron exposure (e^-/^Å^2^) | 45-50 | | | | | |
| Defocus range (μm) | -0.5 to -2.5 | | | | | |
| Pixel size (Å) | 0.664 | | | | | |
| Energy filter (eV) | 20 | | | | | |
| Micrographs (#) | 19301 | 33055 | 12897 | 9318 | 7281 | 5725 |
| Symmetry imposed | C1 | | | | | |
| Particles in final Class | 660276 | 154538 | 733688 | 136896 | 373279 | 133895 |
| Map resolution (Å)  (FSC 0.143)  Map sharpening B factor (Å^2^) | 3.8  -197.74 | 3.9  -168.435 | 3.6  -223.113 | 3.4  -137.7 | 3.6  -148.229 | 4.7  -240.975 |
| Local Resolution Range (Å^2^) | 3.7-4.9 | 3.8-5.9 | 3.5-4.5 | 3.4-5.0 | N/A | N/A |
| **Refinement** | ABCB1_apo_ | ABCB1  _Taxol/ATP_ | ABCB1  _Zosuquidar/ATP_ | ABCB1_ATP𝞬S_ | | |
| Model composition  Non-hydrogen atoms  Protein residues  Ligands | 8664  1087  15 | 9398  1158  24 | 9525  1170  25 | 9128  1157  13 | | |
| *B* factors (Å^2^)  Protein  Ligand | 65.21  41.21 | 128.74  100.83 | 79.80  60.54 | 70.65  62.68 | | |
| R.m.s. deviations  Bond lengths (Å)  Bond angles (°) | 0.003  0.489 | 0.002  0.483 | 0.003  0.516 | 0.004  0.579 | | |
| Validation  MolProbity score  Clashscore  Poor rotamers (%) | 1.84  7.50  0.22 | 1.62  9.52  0.52 | 1.73  6.72  0.31 | 1.59  7.68  0.11 | | |
| Ramachandran plot  Favored (%)  Allowed (%)  Disallowed (%) | 93.52  6.48  0.0 | 97.40  2.52  0.09 | 94.85  4.81  0.34 | 97.05  2.95  0 | | |
