# Supplementary material for: Structural insights into binding-site access and ligand recognition by human ABCB1
Source: EMBO J. 2025 Jan 13;44(4):991–1006. doi: 10.1038/s44318-025-00361-z (PMC11833089; doi:10.1038/s44318-025-00361-z)
Supplement: Supplementary file 3 — Appendix [file 44318_2025_361_MOESM3_ESM.pdf]

APPENDIX for

Structural insights into binding-site access and ligand recognition by human ABCB1

Devanshu Kurre[1], Phuoc X. Dang[1,2], Le T.M. Le[1,3], Varun V. Gadkari[4], and Amer Alam[1#] [1] The Hormel Institute, University of Minnesota, Austin, Minnesota 55912, United States

[2]Current Address: Department of Pharmacy - Inpatient, Mayo Clinic, Rochester, Minnesota 55901, United States

[3]Current Address: Department of Biochemistry and Molecular Biology, Mayo Clinic, Rochester, Minnesota 55901, United States

[4]Department of Chemistry, University of Minnesota, Minneapolis, Minnesota 55455, United States

[#]Address correspondence to: aalam@umn.edu

TABLE OF CONTENTS

Table of Contents 1

Appendix Figure S1. Data processing overview 2

Appendix Figure S2. Data processing overview 3

Appendix Figure S3 EM Density maps for lipid embedded ABCB1 4

Appendix Figure S4. Local Density filtered Maps. 5

Appendix Figures

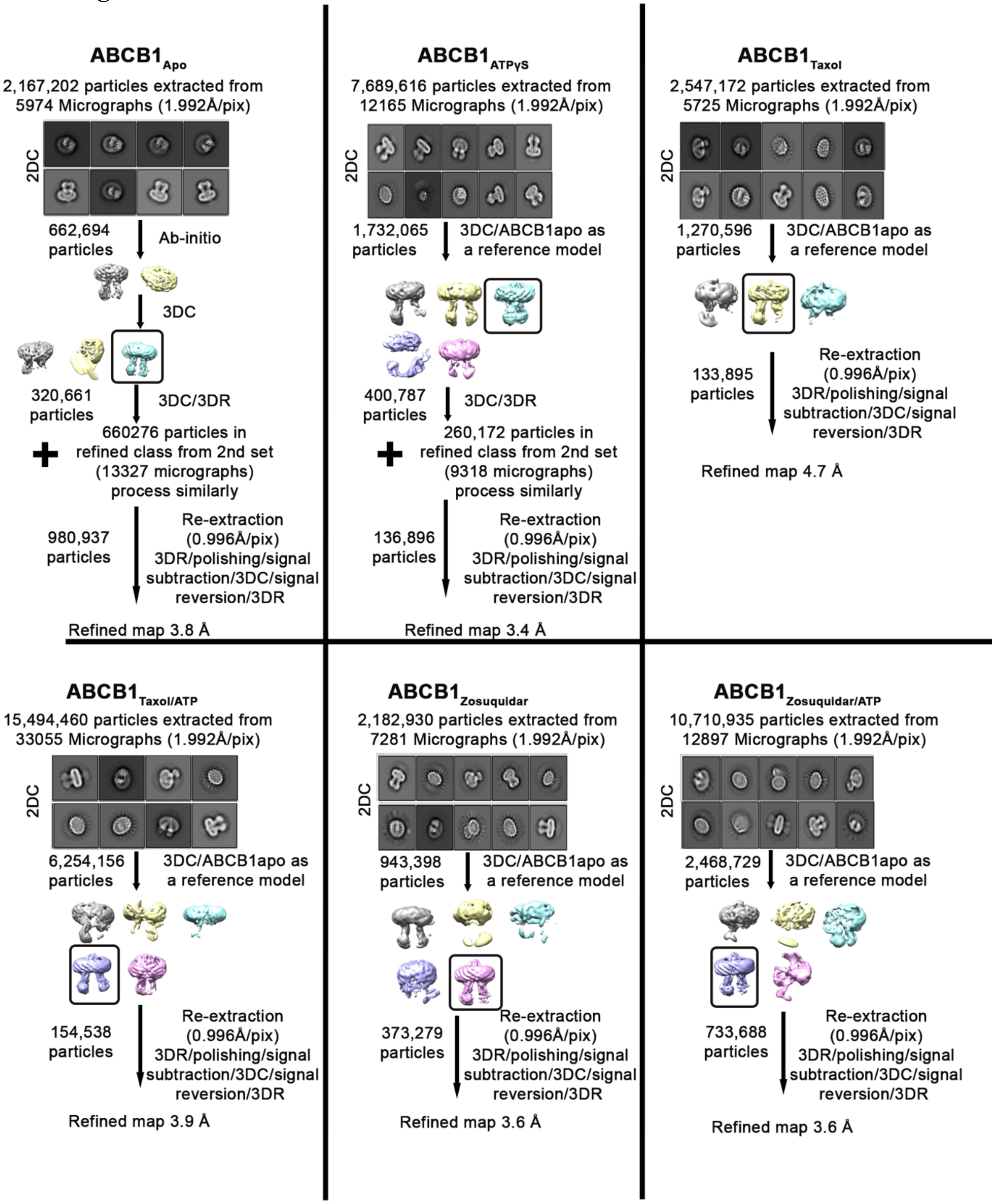

Appendix Figure S1. Data processing overview. 3D classes chosen for further processing are boxed.

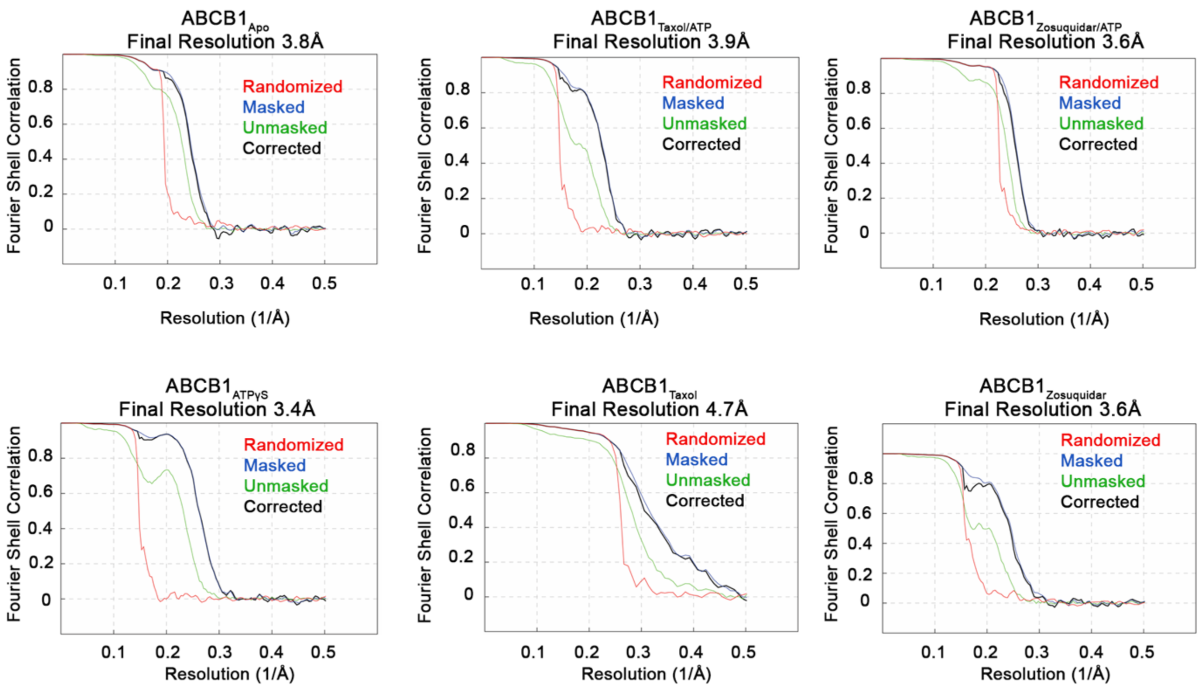

Appendix Figure S2. FSC curves. for final human ABCB1 cryo-EM maps. Final resolutions are reported above each FSC curve.

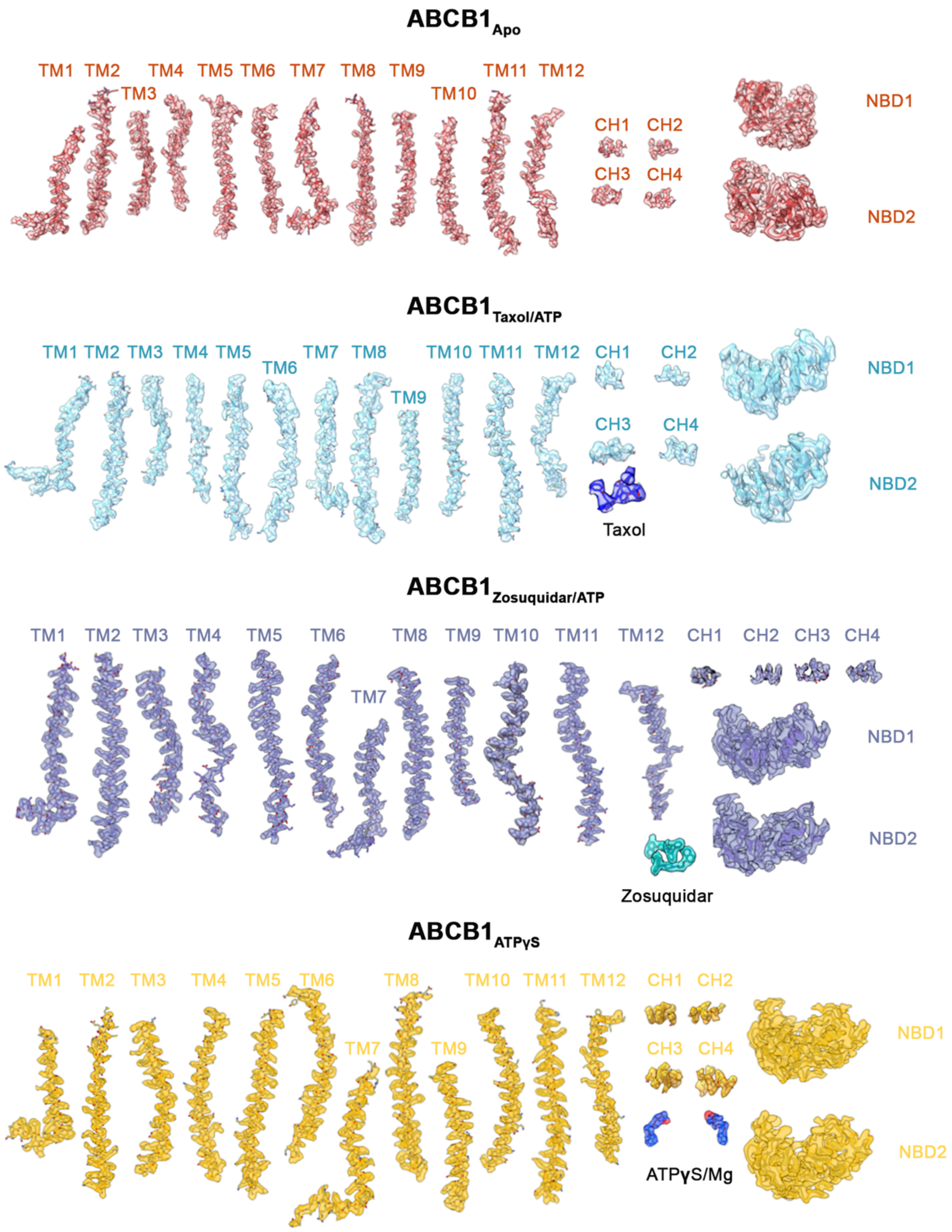

Appendix Figure S3 EM Density maps for lipid embedded ABCB1. Contour levels for Apo: 0.012; Taxol/ATP complex: 0.011; zosuquidar/ATP complex: 0.031; and ATP***γ***S complex: 0.011.

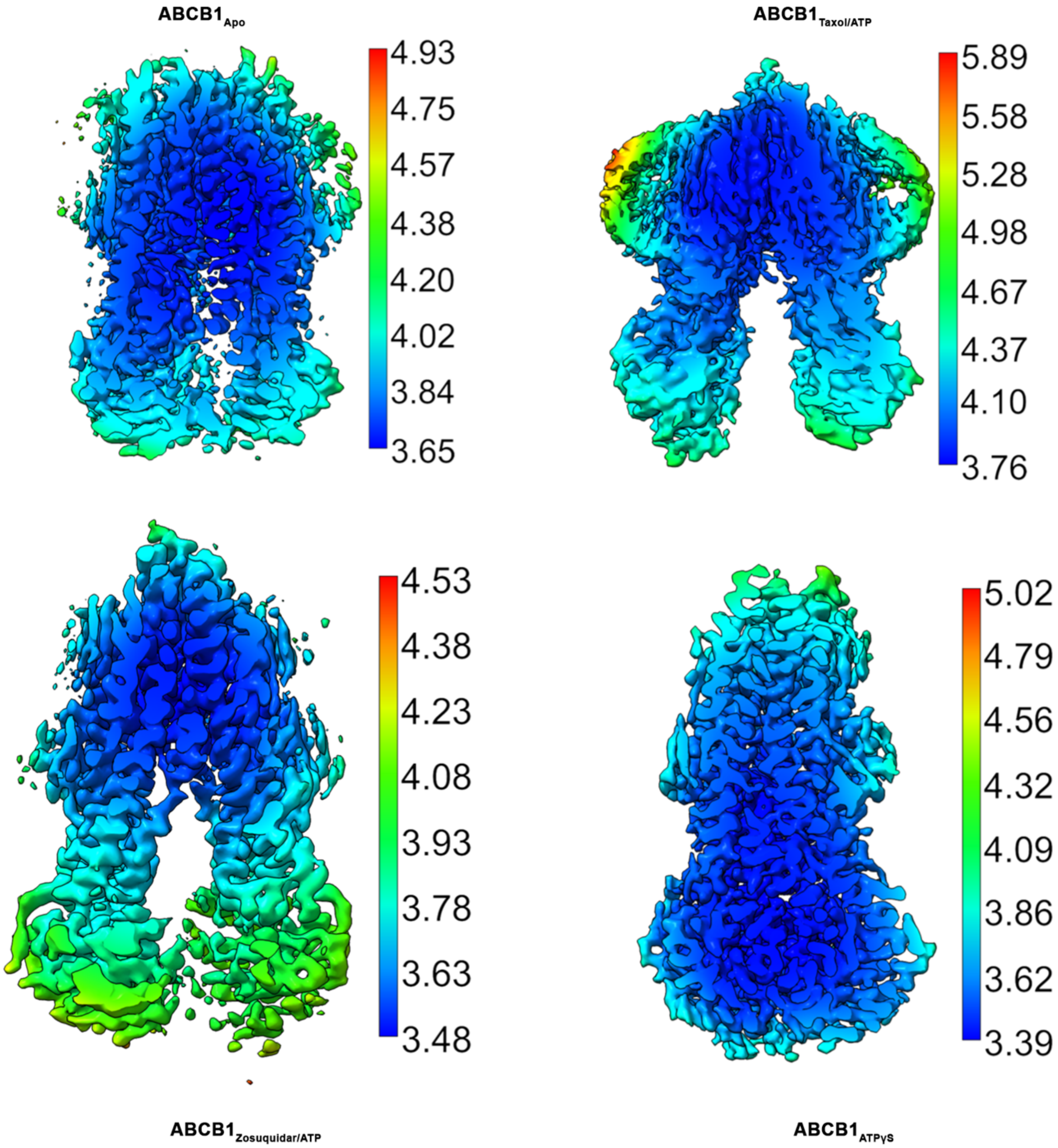

Appendix Figure S4. Local Density filtered Maps. Color Key indicates Resolution range for each filtered map.
