# Supplementary material for: Structural insights into binding-site access and ligand recognition by human ABCB1
Source: EMBO J. 2025 Jan 13;44(4):991–1006. doi: 10.1038/s44318-025-00361-z (PMC11833089; doi:10.1038/s44318-025-00361-z)
Supplement: Supplementary file 6 — Expanded View Figures [file 44318_2025_361_MOESM6_ESM.pdf]

Expanded View Figures

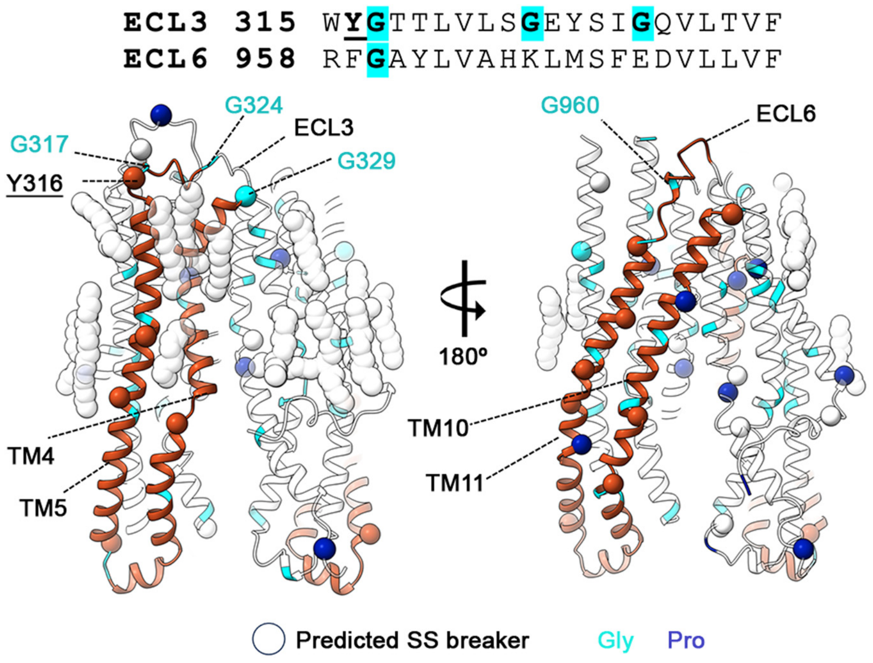

Figure EV1. Secondary structure (SS) breaks in apo-ABCB1.

Gly and Pro residues colored teal and blue, respectively, and predicted SS breaks shown as spheres. An ECL3 and ECL6 sequence alignment is also shown with residues colored similarly and predicted SS-breaking residues underlined. TM4/5 and TM10/11 pairs are colored red. Acyl chains for prospective lipid/sterol molecules are shown as transparent spheres.

 © The Author(s)

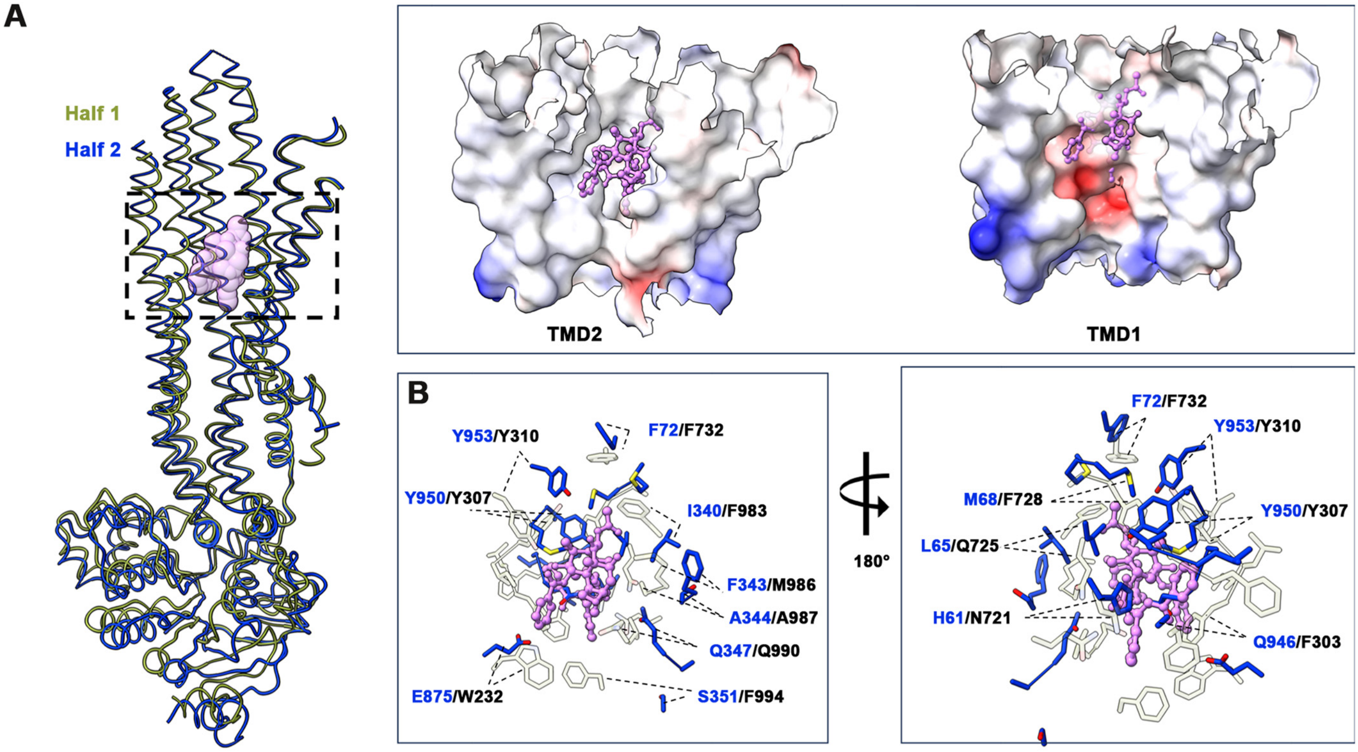

Figure EV2. Mismatch between TMD1 and TMD2 cavities for Taxol binding.

(**A**) Overlay of domain-swapped (DS) halves of ABCB1. The Taxol molecule bound to $TMD2_{DS}$ is shown as transparent pink spheres. The Zoom panel shows electrostatic potential map of the $TMD2_{DS}$ cavity (left) and its $TMD1_{DS}$ cavity equivalent (right) showing electrostatic and steric clashes with Taxol. (**B**) $TMD1_{DS}$ equivalent residues of $TMD2_{DS}$ residues (Blue sticks) within 5 Angstroms of bound Taxol (transparent sticks), with residue labels colored similarly.

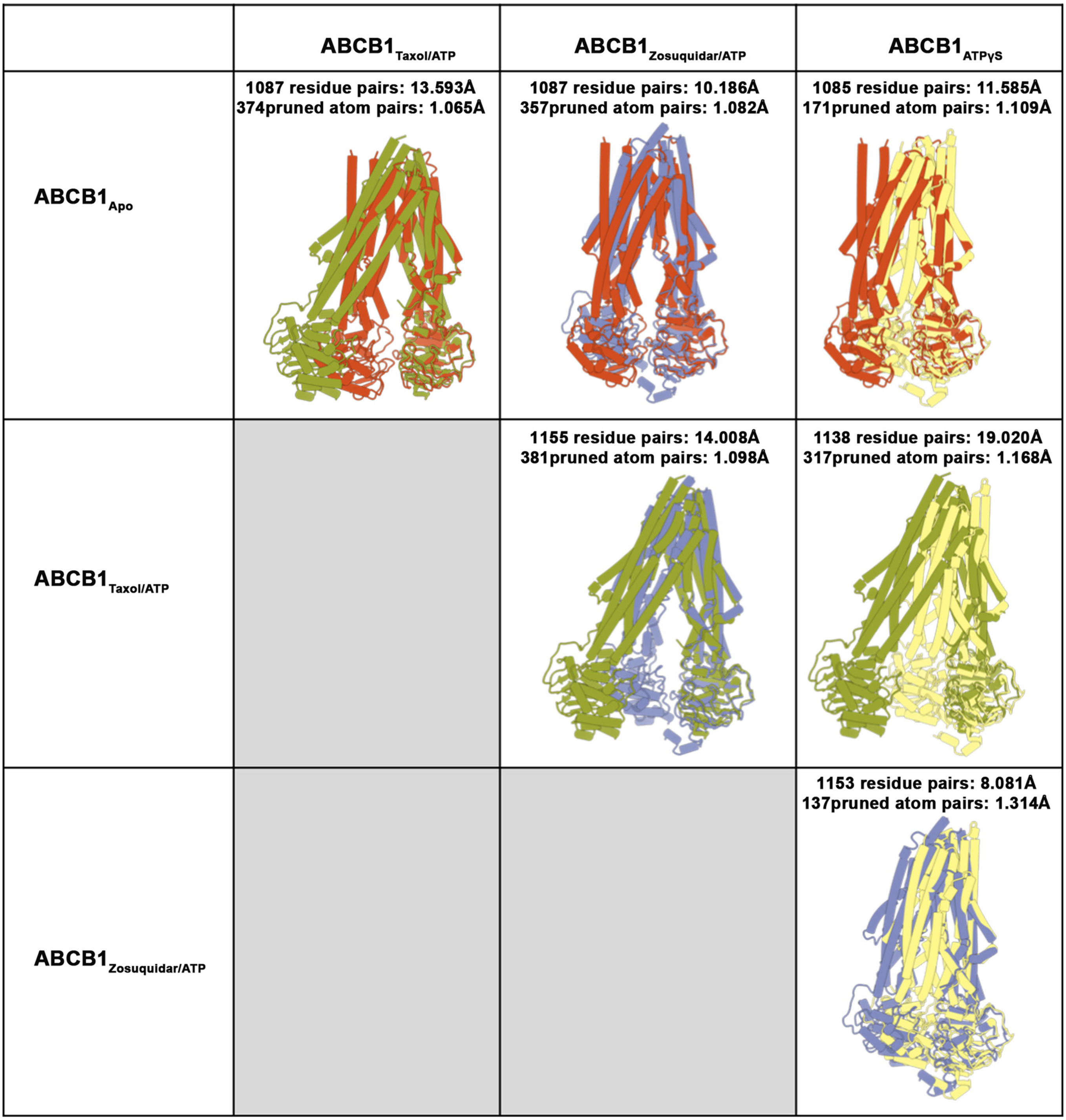

Figure EV3. Overlay of different conformational states of ABCB1.

Overall structural alignments between each conformation. R.m.s.d. values are also shown for total and aligned C alpha pairs.

 © The Author(s)
